# Parasites provide evidence for fish consumption among Iron Age Siberian pastoralists

**DOI:** 10.1038/s41598-024-74284-w

**Published:** 2024-10-09

**Authors:** Sergey Slepchenko, Timur Sadykov, Daria Fomicheva, Jegor Blochin, Gino Caspari

**Affiliations:** 1 Tyumen Scientific Center, Siberian Branch of the Russian Academy of Sciences, 86 Malygina Street, 625003 Tyumen, Russia; 2 Institute for the History of Material Culture, Russian Academy of Sciences, Dvortsovaya Nabereznaya 18, 191186 St. Petersburg, Russia; 3 Domestication and Anthropogenic Evolution Research Group, Max Planck Institute of Geoanthropology, Kahlaische Str. 10, 07745 Jena, Germany; 4 Institute of Archaeological Sciences, University of Bern, Mittelstrasse 43, 3012 Bern, Switzerland

**Keywords:** Parasites, Helminths, Palaeoparasitology, Archaeoparasitology, Pastoralism, Iron Age, Siberia, Eurasia, Anthropology, Archaeology

## Abstract

The subsistence economies of prehistoric pastoralists of the Eurasian steppes have long been viewed through an oversimplified model of reliance on domesticated animals. This conceptualization has begun to shift dramatically through the introduction of scientific analyses, pivoting towards an evidence-based interpretation of economic flexibility and adaptive heterogeneity. Here we provide insights into the dietary practices of Iron Age pastoralists in Siberia through an archaeoparasitological analysis. Soil samples from the Tunnug 1 site in southern Siberia reveal the presence of helminth eggs of *Taenia sp*. (likely), *Trichuris sp*., and *Dibothriocephalus sp*. This indicates that the diet of the analysed prehistoric population might have included beef and did include freshwater fish, occasionally consumed in undercooked or raw form. Despite the primary reliance on pastoralism and possibly small-scale millet agriculture, these populations engaged in diverse dietary practices, including fish consumption. Additionally, the presence of *Trichuris sp.* eggs points to poor sanitary conditions, possible consumption of contaminated plant foods, and the contamination of drinking water with feces. By providing direct evidence of dietary habits, archaeoparasitology complements isotopic analyses and contributes to a more nuanced understanding of the subsistence strategies.

## Introduction

Recent advances in scientific methods and their broader application in the archaeology of the Eurasian steppes have provided nuance to the previously oversimplified conceptualizations of diet and mobility in the Iron Age. Growing scholarly interest in the study of the pastoralist economies of the Eurasian steppe has led to an intensification of archaeological excavations as well as new kinds of data allowing a discussion of subsistence models and social organization of pastoralist societies arriving at more complex models, leaving room for heterogeneity, economic flexibility, and social complexity [cf.^1–4^]. Despite their potential to provide more insights into diet, mobility, health, and hygiene, parasitic infections among prehistoric Eurasian steppe populations have been the topic of only a small number of research papers.

So far, the study of the mummified soft tissues of the pelvic girdle of several individuals from Doge-Baary II burial ground in Tuva, dated to the 5-fourth centuries BCE provided evidence of *Trichuris sp*.^5^. The eggs of intestinal parasites of two genera of *Trichuris sp*. and *Dibothriocephalus sp.* were identified in soil samples from the anterior sacral surface of four individuals from the Early Sarmatian burial mound Kovalevka, dated to the 2-first centuries BCE^6^. Data on parasitic diseases in prehistoric Eurasian pastoralist populations are exceedingly rare because samples for such studies are rarely taken during archaeological excavation and the availability of mummified human remains are limited.

**Fig. 1:**
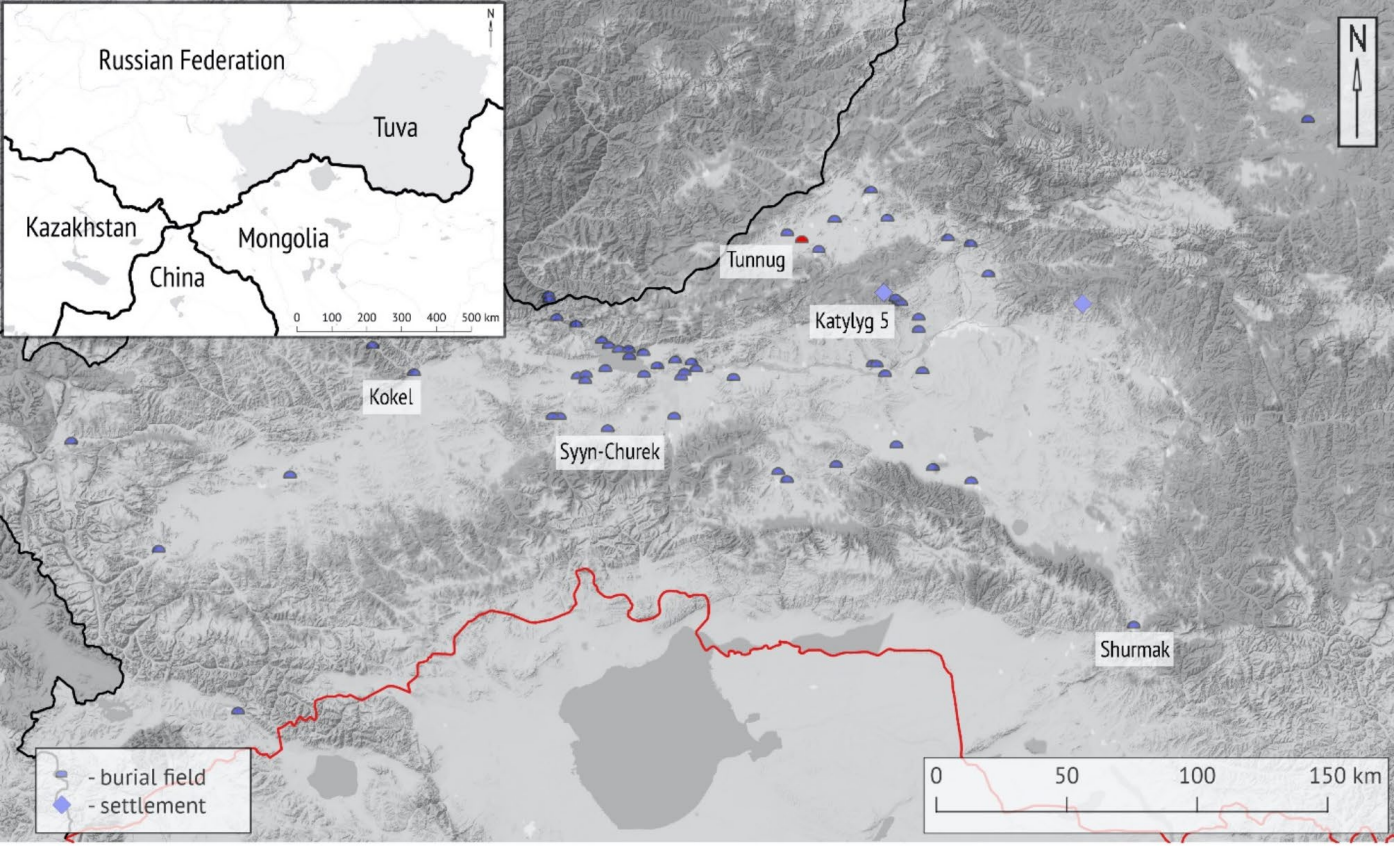
The location of the Tunnug 1 site in the Uyuk Valley (in red), southern Siberia in relation to other contemporary burial sites (purple half circles) and settlement sites (purple squares) of the Iron Age Kokel culture. Map adapted from^9^ originally published under (CC BY 3.0) license.

**Fig. 2:**
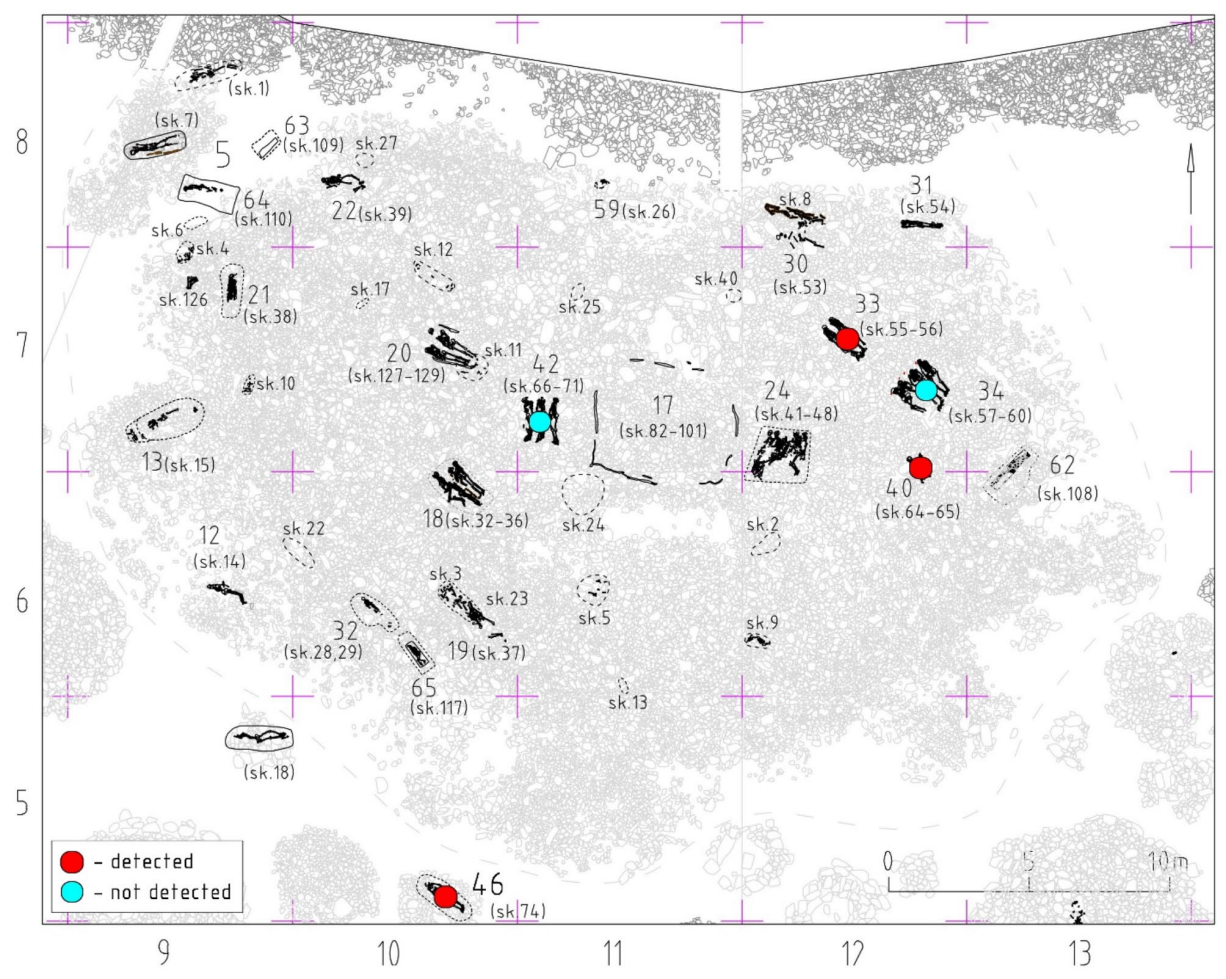
The amorphous barrow of the Kokel culture in the southern periphery of the Tunnug 1 site. Sampled burials are marked with a red (positive) or teal circle (negative). Map adapted from^9^ originally published under (CC BY 3.0) license.

The purpose of this study is to determine the parasitic spectrum among the people of the Kokel culture (3rd–4th c. CE), buried on the periphery of the royal kurgan Tunnug 1 (Fig. 1). The Uyuk Valley, often referred to as the “Valley of the Kings” in Tuva Republic, is famed for its impressive burial mounds and Early Iron Age cultural heritage^7^. The initial excavations in 2017 and 2018 uncovered evidence that the site had been used for funerary purposes for over two millennia^8^. This included funerary ritual activity on the southern periphery of the main burial mound during the Kokel period^9^. In 2019, geophysical surveys revealed a vast periphery surrounding the Early Iron Age burial mound^10^. Over the course of 2018 and 2019, we explored most of the stone structures linked to the Kokel culture, which included smaller over-vessel mounds and an amorphous barrow with a diameter of 28 m. This mound covered dozens of burials and showed signs of long-term ritual continuity (Fig. 2).

The period from the second century BCE to the fifth century CE in Central Asia, known in Soviet archaeology as the "Hunno-Sarmatian Period," is characterized by diverse local cultural phenomena. This period is distinct from both the preceding Early Iron Age and the following medieval cultures. The Kokel culture was first described in the early twentieth century but was only properly defined and classified in the late 1950s^9^. The Kokel culture’s geographic distribution lies mainly within the territory of the Republic of Tuva. Kokel burials typically feature inhumations under mounds, often containing ceramics, iron tools, and animal bones^9^. Settlements like Katylyg 5 suggest a semi-nomadic lifestyle with small fortified communities^11^. Our analysis of the human remains revealed widespread skeletal trauma likely as a result of frequent violent local conflict^12^. A recent isotopic study of carbon, nitrogen, and sulfur from human and animal remains revealed dietary habits and mobility patterns of the Kokel population at Tunnug 1, indicating a diet predominantly based on millet and animal proteins, with minimal differences across gender or social status in funerary practices^13^.

Below we analyze 11 skeletons from five different archaeological structures. All burials are affiliated with the Kokel archaeological culture and date to the second–fifth century CE.

## Results

Archaeoparasitological research material can be divided into two groups based on archaeological context. The first group includes soil samples from contexts which were used over longer periods of time like settlements, latrines, and sewers, containing eggs of human and various animal parasites [cf.^14,15^]. The second group consists of samples from contexts which are the result of a single action in a limited time frame such as the intestinal contents of mummies, human coprolites, and soil samples from undisturbed burials [cf.^16,17^]. The soil samples from the burials of the southern periphery of Tunnug 1 fall into this second group, suggesting a clear human origin for the parasitic eggs found there.

**Fig. 3:**
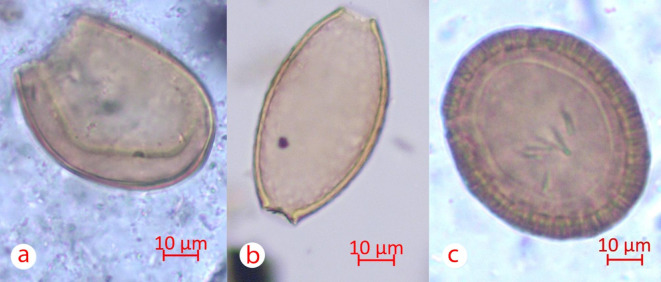
(**a**) *Dibothriocephalus sp*. egg, (**b**) *Trichuris trichiura* egg, (**c**) *Taenia sp*. (?) egg.

Eggs of a characteristic spherical shape and a thick radial striated shell of light brown color were found in 4 out of 11 soil samples taken from the anterior sacral surface and within the foramina (Table 1). Some eggs still have clearly visible hooks. All of our control samples were negative. Based on the available morphological and morphometric data, the eggs found can be attributed to various families (Fig. 3). The dimensional characteristics of the detected parasite eggs are shown in Table 2.

**Table 1:** List of sampled Kokel culture individuals and correlation with individually dated archaeological structures (cf. Fig. 2 for structure location).

Archaeological structure	Individual within the structure	Skeleton code	Biological sex (Milella et al.^12,13^)	Age at death (Milella et al.^12,13^)	Approximate date	Lab code	Result
33	1	55	M	≥ 50 y	CE 251–405 (2σ) (Sadykov et al. 2021)	2720	Taenia sp.
33	2	56	M	35–49 y	Simultaneous with the skeleton 55, same burial structure	2721	Taenia sp
34	1	57	NA	8 ± 2 y	CE 245–376 (2σ) (Sadykov et al. 2021)	2722	–
34	2	58	M	19–34 y	CE 255–408 (2σ) (Sadykov et al. 2021)	2723	–
34	3	59	M	19–34 y	CE 237–348 (2σ) (Sadykov et al. 2021)	2724	–
34	4	60	M	35–49 y	CE 252–406 (2σ) (Sadykov et al. 2021)	2725	–
40	1	64	NA	9 ± 3 y	Planigraphically and archaeologically 3rd–4th c. CE	2726	Taenia sp., Trichuris sp.
42	2	67	NA	≥ 50 y	Simultaneous with the skeleton 68, same archaeological structure	2728	–
42	1	66	F	35–49 y	Simultaneous with the skeleton 68, same archaeological structure	2729	–
42	3	68	M	≥ 50 y	CE 245–376 (2σ) (Sadykov et al. 2021)	2730	–
46	1	74	M	35–49 y	Planigraphically slightly later than the others, belongs to the same archaeological culture 3rd–4th c. CE	2732	Taenia sp., Dibothriocephalus sp.

**Table 2:** Measurements of parasite eggs.

Species	Total number of eggs in the sample/number of eggs measured	Size	
		Egg length in μm	Egg width in μm
		Min—Max (M ± SD)	Min—Max (M ± SD)
Taenia sp. (?)	92/75	44.6–28.2 (37.7 ± 3.22)	39.5–25.6 (33.1 ± 2.5)
Trichuris sp.	2/2	60.2–59.8	33.6–33.5
Dibothriocephalus sp.	1/1	61.8	45

Eggs of intestinal parasites of the family *Taeniidae* were previously found in samples from the anterior sacral surface of human remains in burials. The three species previously found are beef tapeworm (*Taenia saginata*), pork tapeworm (*Taenia solium*) and Asian tapeworm (*Taenia asiatica*)^18^. Cattle is an intermediate host of the beef tapeworm. At the Kokel burials of Tunnug 1, neither pig bones nor cattle bones were found. However, the zooarchaeological record of excavations at the Kokel settlement Katylyg 5 indicates that cattle was key to the diet of people during this time^19^. We can therefore speculate that beef tapeworm (*Taenia* saginata) is the likely source of the discovered eggs.

As for the detection of eggs of nematodes of the genus *Trichuris*, the sampling context and size of the eggs allow us to narrow the species to the human whipworm (*Trichuris trichiura*)^20,21^. In one soil sample taken above the surface of the sacrum of individual 1 from structure 40, eggs were found that were characteristically elongated barrel-shaped with tapering towards the poles and a thick shell with double contour. There were no plugs on the poles of all the eggs. Based on morphological and measurement characteristics, the eggs found belong to nematodes of the genus *Trichuris* (Fig. 3b).

The morphological and morphometric similarity of different eggs of the genus *Dibothriocephalus* make it difficult to determine the species of the egg of this genus. However, on the territory of Southern Siberia, only broad lentensis (*Dibothriocephalus latum*) and gull lentensis (*Dibothriocephalus dendriticum*) are of epidemiological significance, which again allows to narrow down the selection of species^22^. In a soil sample from the surface of the sacrum of individual 1 from structure 46, an oval egg was found, light brown in color with a single-layer dense shell. The lid of the egg was missing. Opposite to the attachment of the operculum, eggs of this type have a visually detectable thickening. The size and morphological features indicate that the detected parasite egg belongs to the tapeworms of the genus *Dibothriocephalus* (Fig. 3a).

## Discussion

The economic complexity of Eurasian steppe populations has garnered increasing interest in archaeology. The rejection of simplistic models, termed “nomadic bias” by Spengler et al.^3^, has led to a robust research agenda over the past decade. Traditional assumptions about these cultures—such as reliance on domesticated animals, low social complexity, and high mobility—are being replaced by perspectives emphasizing their heterogeneity, economic flexibility, and social complexity. In addition to archaeological, archaeozoological, and palaeobotanical investigations, isotopic analyses are continuously providing new insights into the past dietary and mobility patterns of these populations [cf.^23–27^]. Studies of parasites are able to contribute to this discourse and detail the picture, but they are so far rarely conducted on prehistoric sites in the Eurasian steppes.

The Kokel population who created the burials on the southern periphery of the Tunnug 1, had a mixed diet, including a significant portion of proteins from terrestrial animals (through meat and/or dairy products), as well as C4 plants^13^. If the identification of bovine tapeworm (*Taenia saginata*) eggs in soil samples from the sacrum of buried people is correct, this would confirm the presence of cattle meat in the diet. However, we cannot be sure of the identification for now as the only aspect supporting this identification is the archaeozoological record from other Kokel sites^19^.

In addition to beef, according to the analysis of stable isotopes, fish could theoretically be present in the diet of people from Tunnug 1, at least in some individuals. However, due to the fact that the range of δ15N values in modern fish falls within the range of herbivores from Tunnug 1, it is not possible to assess the contribution of freshwater fish to the diet of the population^13: 132^. The presence of a tapeworm egg of the genus *Dibothriocephalus* in structure 46 allows, with a certain degree of caution, to assert the presence of fish in the diet of this population. This reinforces that Eurasian steppe populations included freshwater fish in their diet as reported by other studies^25,28–30^. Interestingly, ethnographic literature indicates that fish consumption is not a particularly common practice among local pastoralists^31: 210–231^. There is no direct evidence of a strict cultural taboo in the area, but fish consumption remains rare today. In the Tuvan language, the common word for fish is “balyk”, however, the most widely used expression is “sug kurtu”, which can be translated as “water worm”^32: 529^. In Tuva, fishing was a practice among some tribes, but it was never a primary occupation and never involved the use of boats^31: 165^. The Uyuk River, which flows in the vicinity of the site, is suitable for fishing, although large fish are not abundant.

Eggs of the helminths Trichuris trichiura are found on a large number of chronologically and geographically different archaeological sites in Eurasia, South and North America and Africa^33^. Infection with this geohelminth is most likely in conditions of poor personal hygiene and in general is evidence of poor sanitary and hygienic conditions. Unlike the above-mentioned biohelminths that clearly indicate the consumption of a particular product, human whipworm eggs can only indirectly indicate nutrition patterns. The eggs of this helminth were previously discovered in the mummified bodies from the Doge-Bary II burial ground, dated to the fifth-fourth centuries BCE^5^. It is thus safe to assume that the parasite had been present in Tuva for an extended period of time. Considering this is the second case of discovery of this geohelminth on the territory of Tuva allows us to raise the question if it was indeed more prevalent on the territory of southern Siberia in the past. The mobility of the Kokel people, judging from the material culture was limited to a regional level^9^. Paleoclimatic reconstructions for the Uyuk valley, where the Tunnug 1 kurgan is located, have been undertaken several times, but still lack sufficient details and the temporal resolution remains low^34,35^. The lifecycle of the geohelminth Trichuris trichiura demands warm and humid conditions of the subtropics and tropics, although it is widespread in temperate zones^20^. In Tuva with its harsh continental climate, it is unlikely that this whipworm could develop. However, even in the generally dry Tuva, there are still some areas with sufficient humidity and warmth. The possibility of local anthropogenic loci of Trichuris trichiura for example in permanent warm dwellings could be considered. Overall, however, presence of this parasite is more linked to the hygiene conditions, the waste management in this prehistoric community, and the pollution of drinking water with feces, rather than with diet or mobility.

## Conclusion

This analysis demonstrates that parasites provide valuable evidence for the dietary habits of Iron Age Siberian pastoralists, particularly regarding their fish consumption which is sometimes difficult to discern in isotopic studies. The discovery of helminth eggs of *Dibothriocephalus sp*., *Trichuris trichiura*, and possibly *Taenia sp*. in soil samples from burials at Tunnug 1 suggests that the diet of these populations might have included beef, did include freshwater fish, and possibly contaminated imported plant foods. This parasitological evidence complements isotopic analyses, revealing a complex subsistence strategy involving both terrestrial and aquatic resources. The findings underscore the importance of integrating palaeoparasitological data into broader archaeological and anthropological research to enhance our understanding of ancient diets, health, and mobility patterns.

## Methods

**Fig. 4:**
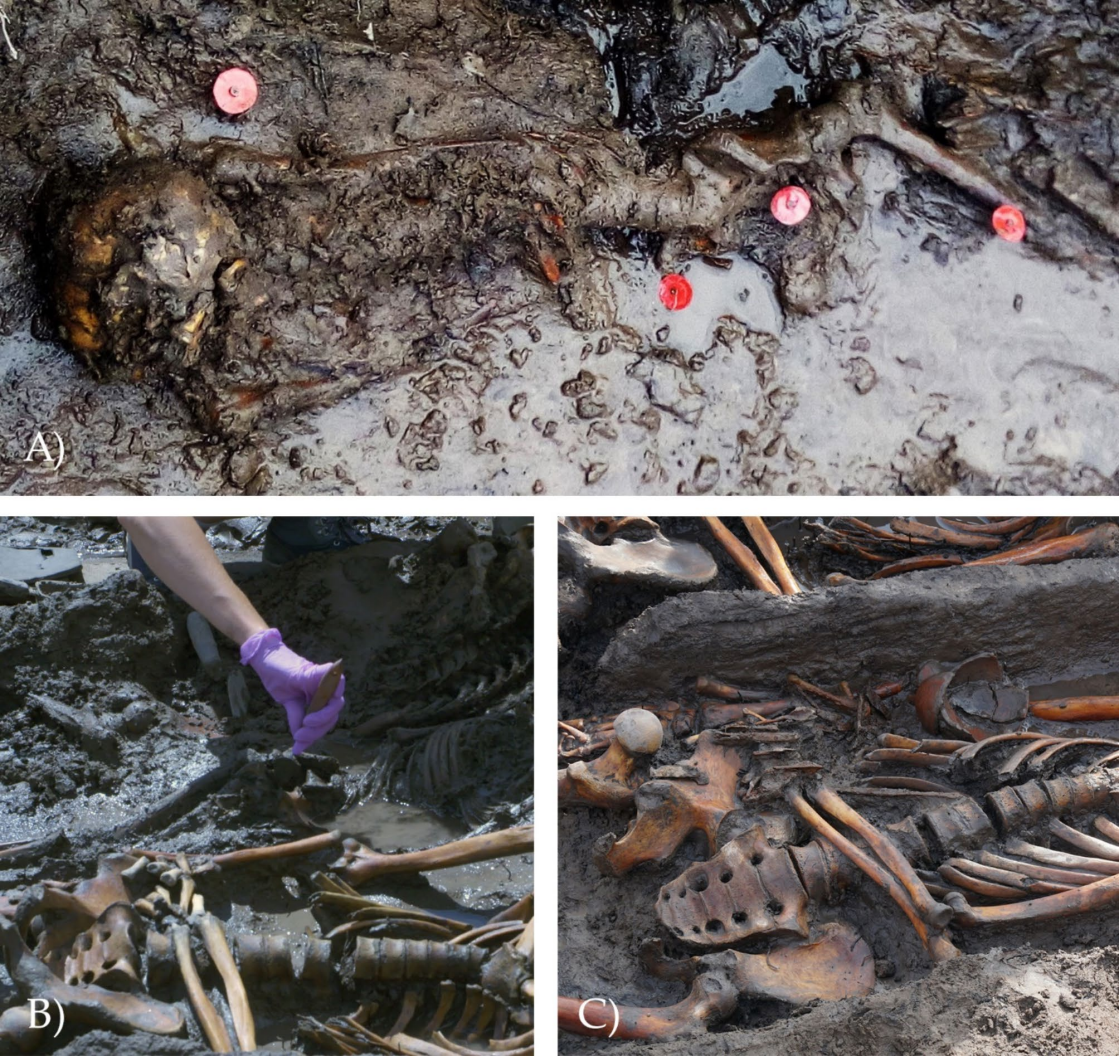
(**A**) Uncleaned burial (structure 33, burial 1) with marked sampling points (Photo Credit: D. Fomicheva). (**B**) The sampling process (Photo Credit: T. Wallace. (**C**) Post-sampling pelvis (Photo Credit: T. Wallace).

The materials this archaeoparasitological study is based on are soil samples taken during the excavation of burials from the anterior sacral surface and within the foramina—1–2 cm of matrix above the sacrum of 11 individuals (Fig. 4). Samples were taken from all burials where contamination-free sampling was possible. Many burials pits on the Tunnug 1 site are located below the groundwater level and do not allow for sampling without contamination. The control samples were taken at the bottom of the grave at the head of the buried individuals. In the case of individual 2 from structure 33, the control sample was taken at a small distance from the femur, since there were fragments of ceramics and the remains of a wooden coffin situated near the skull. In case of individual 1 from structure 46, 2 samples were taken: one between the pelvic bones and one directly above the sacrum. Archaeoparasitological data were obtained from both and the results were combined.

Further processing of the samples was carried out in the laboratory [cf.^36^]. A 0.5% solution of trisodium phosphate (Na_3_PO_4_) was added to dry soil samples weighing from 17 to 112 g placed in a beaker with a volume of 800 ml. Chemical cups with samples were covered with filter paper. Over the course of 8 days, the colored filler solution of 0.5% trisodium phosphate was replaced three times while stirring the content each time. After the filler fluid stopped dyeing, it was removed. The sediment was washed with distilled water until a neutral medium was reached. At the next stage, the contents of the glass were passed through a sieve with a diameter of 200 µm, mineral and organic fragments larger than this size were removed. The filtered solution with an organic fraction and a finely dispersed mineral component was poured from a bowl into a glass. The filler liquid was drained, the substrate was centrifuged for 15 min at 1500 rpm in 50 ml tubes and dried. Due to the fact that the substrate contained a significant amount of fine sand, which interferes with the preparation of specimens and microscopy, we used hydrofluoric acid (49%) to remove it. A 10% HCl solution was added to dissolve colloidal SiO2 and silicofluorides. Acid residues were washed by repeated centrifugation with distilled water. The pH level was controlled with litmus paper. The sediment was then passed through a 10 µm sieve. The fraction remaining on the sieve was collected in 10 ml conical tubes into to which glycerin was added. The contents of the tube were stirred and heated in a water bath at 80° C for 10 min. Then the tubes were centrifuged for 5 min at 1500 rpm, and the glycerin was drained. 20 micro-preparations were prepared from each sample, which were analysed using AxioSkop 40 and MicMed 2 var.2 microscopes with a magnification of 100× and 400×. The software AxioVision 4.6 and Scope Photo 3.0 were used for the size measurement. The indicators of the absolute size range (minimum, min; maximum, max), arithmetic mean (mean, M) with standard deviation (SD) were used as descriptive statistics for the measured characteristics of eggs. The MS Excel program from MS Office 2019 was used to calculate numerical parameters and plot diagrams. To determine the species of human parasite eggs, the guidelines by Ash and Orihel^37^ and Gayevskaya^38^, as well as materials from the CDC DPDx—Laboratory Identification of Parasites of Public Health Concern^39^ were used.

## Data Availability

All data are contained within the manuscript.
